# Psychedelic Assisted Psychotherapy preparing your target using psychohistoriography: a Jamaican perspective

**DOI:** 10.3389/fpsyt.2023.1136990

**Published:** 2023-06-29

**Authors:** Winston De La Haye, Geoffrey Walcott, Jordan Eaton, Jhoelle Beckford, Janelle Greene

**Affiliations:** ^1^Department of Community Health and Psychiatry, The University of the West Indies, Kingston, Jamaica; ^2^Caribbean Institute of Mental Health and Substance Abuse, The University of the West Indies, Kingston, Jamaica; ^3^Kingston Public Hospital, Kingston, Jamaica; ^4^Department of Sociology, Psychology and Social Work, The University of the West Indies, Kingston, Jamaica

**Keywords:** psilocybin, psychedelics, Psychedelic Assisted Psychotherapy, psychohistoriography, new frontier

## Abstract

The efficacy of psilocybin and other psychedelics as modes of treatment have been demonstrated through clinical trials and other studies in the management of a number of mental illnesses, including some treatment resistant cases. In Psychedelic Assisted Psychotherapy (PAP), psychedelics catalyze or enhance the experience fostered by psychotherapeutic methods. Psychohistoriographic Brief Psychotherapy, conceptualized by the late Professor Frederick Hickling in the 1970′s in Kingston, Jamaica, offers a pathway for exploration in the Jamaican context. Applied to individuals, Psychohistoriographic Brief Therapy (PBT) has already shown success in patients with personality disorders in Jamaica through a process which includes documenting life experiences in a psychohistoriogram. In the De La Haye psilocybin Treatment Protocol (DPTP), micro-doses of crushed, dried psilocybin mushrooms are taken throughout an 8-week outpatient process of documenting the components of the psychohistoriogram, making use of the increased openness and empathy associated with the use of psychedelic agents. These sessions are followed by supervised in-office therapeutic/mystical doses of crushed, dried psilocybin mushrooms in the 9th week. Given the legal status and availability of psilocybin containing products in a few countries like Jamaica, there is a potential role for a regulated psychedelic industry contributing to the body of useful and rigorous clinical research which is needed in this area. Clients could benefit as we venture into this new frontier in psychiatry.

## Introduction

Psychedelic Assisted Psychotherapy (PAP), the new frontier in psychiatry (1) has seen a resurgence in interest over the last two decades and is poised for further growth in the treatment of mental illnesses (2, 3). Numerous studies have demonstrated the clinical efficacy of psychedelics in the effective management of treatment resistant depression (4–9), post-traumatic stress disorder (10, 11), anxiety disorder (12), substance use disorder (13, 14), obsessive-compulsive disorders (15), and existential distress (7, 16). With demonstrable effects in varying patient populations, there has been remarkable renewed promise for the use of psychedelics in the treatment of mental illnesses (17). Psychedelics facilitate increased interconnectivity within the brain, a concept referred to as the “entropic brain” by Carhart-Harris (18, 19), while down regulating adverse and traumatic experiences, creating new neural connections and “resetting” the brain (18, 19).

While the active psychedelic compound psilocin was originally isolated in 1958, the use of psychedelics has a long history. Mesoamerican cultures referred to psychedelic rituals before the arrival of Columbus (20, 21). Psychedelics related research was dramatically and hastily discontinued following concerns about recreational drug use and the rise of the “counterculture movement” in the United States of America (U.S.A) (22). This led to their subsequent classification as Schedule I compounds, which are substances with a high potential for abuse and no accepted medical use (23, 24).

Promising results from recent research have led researchers to explore the phenomenon of psychedelics used in conjunction with the utilization of various psychotherapeutic techniques, giving rise to the term Psychedelic Assisted Psychotherapy (PAP). A few studies have shown that PAP may be effective, even for patients considered “treatment resistant” (25). Varying psychotherapeutic modalities have been employed to provide psychological support comprising non-directive preparation, support, and integration, via both individual and group counseling (26–29). An increased focus on identifying ideal modalities for and frequency of psychotherapeutic interventions may help improve outcomes with PAP. While the exact mechanisms by which psilocybin and other psychedelics augment the psychotherapeutic experience are still unclear, it is thought that they catalyze or enhance psychotherapeutic processes (30).

One such psychotherapeutic modality, Psychohistoriographic Brief Psychotherapy, was conceptualized by the late Professor Frederick Hickling in the 1970′s at the Bellevue Mental Hospital in Kingston, Jamaica (31). Historiograpy is the philosophy of methodology of history. Psychohistoriography is a post-colonial, Caribbean model of psychoanalysis and psychotherapy for groups and individuals that addresses multiculturalism, conflict resolution, insight, and social change. It is a psychological method devised for the promotion of insight in psychotherapy, using history as the engine of growth in the therapeutic milieu. Psychohistoriographic Brief Psychotherapy was developed around a technique of life-history mapping, where individual memories, including significant life events are documented around a timeline within a dialectic matrix called the psychohistoriogram. These dialectic antipodes include family events (mother & father), social learning (education & environmental), activity life events (religion and work), cultural life events (extracurricular & artistic/creative work), affect shaping life events (political and emotional), and psychosexual life events (sexual and social). The patient charts these historical facts in line with the associated life parameter. Psychohistoriographic Brief Psychotherapy grew out of the large group technique of Psychohistoriographic Cultural Therapy. It is grounded in the dialectic historical experience of the Caribbean and Caribbean people (31–33). This exploration of memories and events over time, guided by the therapist results in the creation of a poetic script that captures the insight and the journey in arriving at said insight in a tangible form that can be documented for reflection. The insight, described as the psychic centrality, was best captured by Hickling when he wrote, “Psychic centrality refers to a sense of psychological containment or organization of diverse individual points of view through creating a historical map of collective experience” (34). The Psychohistoriographic Brief Psychotherapy model has had success in patients with personality disorders in Jamaica (35). Hickling's (35) paper presents results from evaluations conducted by the therapist himself with no control group or external evaluation of the results.

## The Jamaican setting

Jamaicans have a long history of plant products being readily accepted and preferred for treating various medical conditions (36–39). Historical records and current estimates suggest that this preference and willingness to rely on traditional healing modalities, including herbal remedies for treating medical conditions has continued in our culture for many years (40). In Jamaica, the Dangerous Drugs Act of 1996 and subsequent revision in 2015 make specific stipulations for raw opium and cocoa leaves, prepared opium, cannabis and cannabis containing compounds, and derivates specifically Cocaine and Morphine. There is no current legislative framework governing the possession or use of psilocybin or products containing psilocybin within Jamaica, despite Jamaica being a signatory to the 1961 Single Convention on Narcotic Drugs of 1961, and the subsequent Convention on Psychotrophic Substance of 1971, since 6th October 1989.

In Jamaica patients with mental health conditions requiring specialist care may be referred by their family physician to private Psychiatrists, though some patients also present and are seen without referrals. In the last 12 months, adult patients voluntarily presenting to the first author's private psychiatric practice setting in Kingston, Jamaica have been provided with the option of using psilocybin products for the treatment of major depressive disorder, post-traumatic stress disorder, substance use disorders and existential distress. This has been a welcomed addition to the treatment options being provided in this private psychiatric setting, which also provides conventional/traditional forms of pharmacological treatment of these conditions.

**Figure 1:**
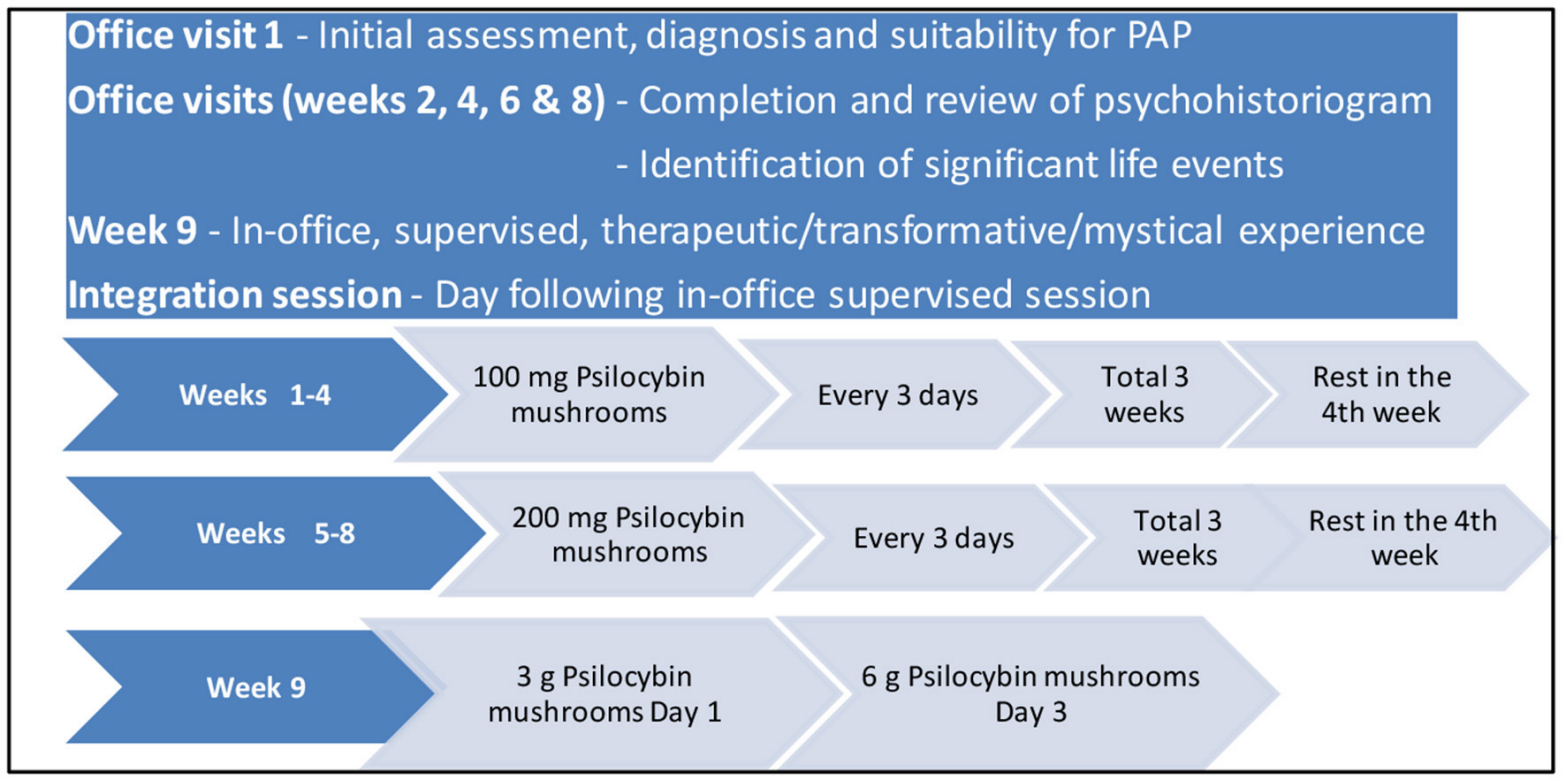
The De La Haye psilocybin Treatment Protocol (DPTP).

Adult patients participate in individual PAP sessions with the exploration of personal memories and significant life events, documenting these in the aforementioned matrix called the psychohistoriogram, making use of the increased openness and empathy associated with the use of psychedelic agents (41–43). Patients are introduced to the De La Haye psilocybin Treatment Protocol (Figure 1), with the option of commencing microdoses of capsule containing crushed psilocybin containing mushroom products on an outpatient basis, escalating to optional in office, supervised, individual therapeutic/transformative/mystical experience doses. Over 40 patients have been managed with this method, with the results/outcomes of these being collated for publication. This number of patients includes some who were previously being managed on conventional antidepressants, diagnosed as treatment resistant, but also some who indicated that they wanted to try this alternative method of treatment for indicated conditions, as they hoped to feel even better than they had been, based on their own research findings on the potential effects of psilocybin in the treatment of some mental health conditions. One patient expressed her hope to at least be able to cut down on her antidepressant dosage as she “does not feel real”.

These patients are being followed up and have all expressed their satisfaction with having the opportunity to access this option of treatment locally, and in a legal, private psychiatric treatment facility. They have also reported their preference for having one-on-one treatment in a private setting of this nature, as opposed to having to share personal experiences in a group type retreat setting, a format which has also been available in Jamaica. Many have expressed their willingness to encourage others to participate in this protocol, where indicated, but have also expressed their concern that products like psilocybin are not available to the average patient who may be unable to participate in private psychiatric management.

## The De La Haye psilocybin Treatment Protocol

The De La Haye psilocybin Treatment Protocol (DPTP) (Figure 1), developed by addiction psychiatrist, Dr. Winston De La Haye consists of a 9-week process of transitioning from out-patient microdoses to in-office, supervised therapeutic/transformative/mystical experience doses of crushed, dried psilocybin mushrooms. Microdoses (100 mg capsules) of crushed, dried psilocybin mushrooms are taken once every 3 days (2 rest days between doses) throughout weeks 1–3 of the 8-week outpatient first phase of the protocol. A 1-week break is then taken (week 4), after which 200 mg microdoses (2 × 100 mg capsules) of crushed, dried psilocybin mushrooms are taken, once every 3 days throughout weeks 5–7. This is followed by a further 1-week break (week 8), which completes the first 8 weeks, first phase of the DPTP protocol where microdoses of crushed, dried psilocybin mushrooms are taken on an out-patient basis. In this 8-week microdose, first phase of the DPTP documenting the components of the Psychohistoriogram are completed and reviewed with the patient. The optional second phase of the protocol consists of the supervised, in-office administration of higher therapeutic/transformative/mystical doses (3 or 6 g) of crushed, dried psilocybin mushrooms (1 g per chocolate bar) on days 1 and 3 of the 9th week of the program. An in-office session on the day following the therapeutic/transformative/mystical dose administration completes the integration sessions, where a revisit of the psychohistoriogram is a significant component.

At their first in-office session, patients present a thorough history and are assessed to determine their diagnosis and suitability for PAP (Figure 1). Those desirous of using this treatment modality are introduced to the concept of Psychohistoriography and guided over the next 2 visits in how to document their histories in the different domains discussed above. Patients commence the out-patient microdose component of the DPTP and are followed up with in-office visits at weeks 2, 4, 6, and 8, as per the DPP (Figure 1). These follow-up sessions include a review of their psychohistoriogram (completed or ongoing), with identification by the therapist of challenging or traumatic life events, documented or reported in their history. In these sessions, patients are provided with continued information on their psilocybin dosages and decisions surrounding continuation in week 9 with a supervised, in-patient mystical/therapeutic dose of crushed, dried psilocybin mushrooms (3 or 6 g). They are also advised of the possibility of any highlighted challenging/stressful/traumatic events they have documented being a part of their mystical/therapeutic dose psilocybin experience. Patients also report in these follow-up sessions on any adjustment in their presenting symptoms. Those agreeing to participate in the 6 h. (9:00 a.m.−3:00 p.m.) supervised session are seen in-office the day following their high dose experience for a specific integration session. Patients are then followed up on a monthly basis, as necessary.

Psychedelic Assisted Psychotherapy, utilizing the Psychohistoriogram as outlined above, prepares a valuable “target”, based on personal memories and significant life events for focus during the administration of therapeutic/transformative/mystical doses of crushed, dried psilocybin mushrooms, facilitating the associated behavioral transformation process resulting fom PAP. Life events charted in the two-dimensional sequence of time and life parameters can be very useful in integration sessions. The crushed, dried psilocybin mushrooms used in the DPTP protocol above are derived from the Psilocybe cubensis species of psychedelic mushrooms, belonging to the fungus family Hymenogastraceae. Psilocybe cubensis mushrooms contain 10–12 mg of psilocybin per gram of dried mushrooms (44). The 8-week microdose period, in addition to facilitating the documentation of the patient's psychohistoriography, also allows for the gradual reduction and eventual discontinuation of antidepressant pharmacotherapy in treatment resistant patients, in addition to facilitating the patient's experience with a new product.

These are interesting times for PAP in the Caribbean in general and Jamaica in particular, benefiting clients as we venture into this new frontier in psychiatry, both in clinical practice and research. We must ensure that the Caribbean's rapidly expanding psychedelic wellness and medical programs are safe, while maintaining the highest ethical standards in the therapeutic use of psychedelics. Given the legal status and availability of psilocybin containing products in a few countries like Jamaica, there is a potential role for a regulated psychedelic industry contributing to the body of useful and rigorous clinical research which is needed in this area.

## Author contributions

WD developed the treatment protocol outlined in the manuscript. WD and GW contributed to the conception of the paper. JG and WD wrote the first draft of the manuscript. All authors wrote sections of the manuscript, reviewed the manuscript, contributed to its revision, and approved the submitted version.
